# Impact of Prolonged Impella 5.5 Support on Post‐Transplant Outcomes: An Institutional Study

**DOI:** 10.1111/ctr.70452

**Published:** 2026-02-25

**Authors:** David Rekhtman, Amit Iyengar, Cindy Song, Michaela Asher, Max Shin, Michael Catalano, Omar Toubat, Emma Morganroth, Alyson Brown, Joyce Wald, Aditya Parikh, Mauer Biscotti, Marisa Cevasco

**Affiliations:** ^1^ Division of Cardiovascular Surgery Hospital of the University of Pennsylvania Philadelphia Pennsylvania USA; ^2^ Division of Cardiovascular Medicine Hospital of the University of Pennsylvania Philadelphia Pennsylvania USA

**Keywords:** heart failure, heart transplantation, temporary mechanical circulatory support

## Abstract

**Objectives:**

Microaxial flow pump devices are utilized as a bridge to heart transplantation for patients in advanced cardiogenic shock. Little is known about the impact of device duration on post‐transplant outcomes. This study aims to compare post‐transplantation outcomes based on duration of support with the Impella 5.5.

**Methods:**

All patients who were successfully bridged to transplant on the Impella 5.5 platform were included and stratified based on support duration (≤ 14 days vs. > 14 days). Baseline clinical characteristics were collected throughout the index admission. Outcomes included 1‐year mortality, complications during the index admission, graft rejection, and rehospitalization within the first year.

**Results:**

Of the 72 patients successfully bridged to transplant, 64% (n = 46) were supported for more than 14 days. When stratified by duration of Impella 5.5 use, there were no differences in pretransplantation clinical status. Length of stay (26 vs. 20 days, *p* = 0.316) and rates of home discharges (65% vs. 67%, *p* = 0.524) were similar. Despite a high prevalence of rehospitalization at 1 year (75%), the 1‐year survival was 96% and similar between the two cohorts (*p* = 0.289).

**Conclusions:**

Use of a prolonged microaxial flow pump may be a safe bridging strategy for heart transplantation given comparable post‐transplant outcomes for patients stratified by support duration.

**Abbreviations**
DSA: donor‐specific antibodies
ECMO: extracorporeal membrane oxygenation
GI: gastrointestinal
HLA: human leukocyte antigen
ICU: intensive care unit
ISHLT: International Society for Heart and Lung Transplantation
LVAD: left ventricular assist device
PGD: primary graft dysfunction
POD: postoperative day
PRA: panel reactive antibodies
tMCS: temporary mechanical circulatory support

## 1 Introduction

Use of temporary mechanical circulatory support (tMCS) has become increasingly common for patients undergoing cardiac surgery [1, 2]. These devices increase cardiac output and systemic perfusion while decreasing ventricular work [3]. The Impella 5.5 (Abiomed) is the latest microaxial flow pump device that was approved for therapeutic use for up to 14 days [4]. Today, it is frequently utilized as a bridge to heart transplantation in patients with cardiogenic shock [1]. Survival at 1 year has previously been reported to be as high as 90%, even though the use of percutaneous tMCS is a known predictor of post‐transplant mortality [2, 5].

As clinical teams become more familiar with managing patients on tMCS, there has been a trend toward prolonged duration of use. Our institution's early experience showed a median duration of 19 days in the first 40 patients [6]. Since then, there have been other institutional studies demonstrating Impella support exceeding 100 days [7]. Efforts to better understand the complication profile associated with prolonged use have resulted in multiple publications focused on thromboembolic events, infections, and local neurovascular injury [6, 7, 8]. However, modern percutaneous devices allow for patient mobilization to prevent the morbidity associated with deconditioning [9].

The impact of prolonged Impella 5.5 support on post‐transplant outcomes is poorly understood. Promising high‐level results have been reported previously by Hong et al. using a national registry, which showed comparable survival and complication rates following transplant irrespective of Impella support duration [10]. However, clinical granularity such as preoperative status, immediate postoperative recovery, and long‐term morbidity is not well captured in large registries, and therefore institutional experience can shed additional light on post‐transplant outcomes for patients bridged to transplant with the Impella 5.5.

## 2 Methods

### 2.1 Patient Selection and Management

We performed a retrospective cohort study of patients at our tertiary‐referral academic medical center supported with the Impella 5.5 and subsequently bridged to heart transplantation (December 2021–December 2024). Patients with devices placed at outside hospitals were excluded. Patients were then stratified into two cohorts depending on the duration of Impella 5.5 support (≤ 14 days vs. > 14 days).

Patients were managed on a step‐down floor after a period of stabilization in an intensive care unit (ICU). Patients were maintained on systemic anticoagulation—either heparin or bivalirudin—to a target aPTT of 60–75 and monitored closely with neurovascular checks, daily labs, and serial echocardiography while on Impella 5.5 support. Patients were encouraged to ambulate and worked to maintain functional status.

Immunosuppression induction consisted of mycophenolate as well as a dose of methylprednisolone, which was determined based on patient comorbidities. Deviation from this standard clinical pathway was seen in a few select cases, primarily due to concomitant kidney transplantation.

### 2.2 Data Collection

This study was exempt from review by the institutional review board at our institution, which waived the requirement for patient consent given all patient data was deidentified (IRB #854461). The institutional dataset was prospectively generated and retrospectively maintained. Baseline characteristics and serologic, hemodynamic, and echocardiographic variables were collected. The primary outcome of interest was 1‐year mortality. Secondary outcomes included complications during the index admission, graft rejection, and causes of rehospitalizations. Graft rejection was determined by the International Society for Heart and Lung Transplantation (ISHLT) histological grade on myocardial biopsy [11].

Pre‐ and postoperative inotrope and vasopressor use was quantified using the vasoactive inotropic score: dopamine (ug/kg/min) + dobutamine (µg/kg/min) + (10 × milrinone [µg/kg/min]) + (10 000 × vasopressin [U/kg/min]) + (100 × epinephrine [µg/kg/min]) + (100 × norepinephrine [µg/kg/min]) [12]. Pre‐ and postoperative transthoracic echocardiography were utilized to assess cardiac function. Intraoperative transesophageal echocardiography was utilized for assessment on postoperative day (POD) 0. Hemodynamics were collected from right heart catheterizations.

### 2.3 Statistical Analysis

All statistical analysis was conducted on Stata/BC 17.0 (College Station, TX). Categorical variables were compared using the Chi‐squared test or Fisher's exact test and presented as *n* (%), while continuous variables were compared with the Kruskal–Wallis test and presented as median [interquartile interval]. Time‐dependent analysis was performed utilizing the log‐rank test and presented as Kaplan–Meier graphs. A *p* value < 0.05 was considered statistically significant for all analyses. Missingness for all variables is presented in Table S1.

## 3 Results

### 3.1 Patient Selection

At the time of analysis, 240 patients had been supported with the Impella 5.5 at our institution, of which 72 (30%) were transplanted. The remaining patients either received a durable left ventricular assist device (LVAD) (62, 26%), died (50, 21%), recovered without the necessity for support (39, 16%), or transferred to an alternative institution due to patient preference (17, 7%) (Figure S1). The median duration of support was 15 days (IQI 7–25 days) with a range from 0 to 134 days on support.

### 3.2 Baseline Characteristics

The Impella 5.5 was used as a successful bridge to transplant in 72 patients at our institution (median: 22 days). There were 26 patients (36%) on Impella 5.5 support for ≤ 14 days, while the remaining 46 patients (64%) were on support for > 14 days, with the longest being 134 days. Baseline patient characteristics are presented in Table 1. The cohorts stratified by duration of support had similar age (49 vs. 53, *p* = 0.361), sex (81% vs. 91% male, *p* = 0.194), and size (28.6 vs. 27.3 kg/m^2^, *p* = 0.897). The etiology of heart failure, INTERMACS class, SCAI class, and comorbidities were also similar between the two groups (all *p* > 0.05). Preoperative sensitization as measured by panel reactive antibodies (PRA) was not different between groups (*p* = 0.585). Concomitant extracorporeal membrane oxygenation (ECMO) support was required for 3 patients (12%) in the ≤ 14 days cohort and 10 patients (22%) in the > 14 days cohort (*p* = 0.280). ECMO was the only concomitant support used in this cohort; one patient required an RVAD, and seven patients were supported with intra‐aortic balloon pumps prior to implantation of the Impella 5.5.

**TABLE 1:** Baseline characteristics.

Variable	Total (*n* = 72)	5.5 ≤ 14 days (n = 26)	5.5 > 14 days (*n* = 46)	*p* value
Age at transplant	52.3 (41.0–59.8)	48.9 (40.0–59.9)	53.0 (46.3–59.7)	0.361
Male sex	63 (87.50)	21 (80.77)	42 (91.30)	0.194
Race				0.194
Asian	2 (2.78)	0 (0.00)	2 (4.35)	
Black	22 (30.56)	5 (19.23)	17 (36.96)	
White	44 (61.11)	20 (76.92)	24 (52.17)	
Other	4 (5.56)	1 (3.85)	3 (6.52)	
Body mass index (kg/m^2^)	28.1 (25.2–30.9)	28.6 (23.7–30.9)	27.3 (25.4–31.2)	0.897
Blood type				0.001
A	24 (33.33)	16 (61.54)	8 (17.39)	
B	14 (19.44)	3 (11.54)	11 (23.91)	
AB	2 (2.78)	1 (3.85)	1 (2.17)	
O	32 (44.44)	6 (23.08)	26 (56.52)	
INTERMACS Class				0.836
1	4 (5.56)	2 (7.69)	2 (4.35)	
2	65 (90.28)	23 (88.46)	42 (91.30)	
3	3 (4.17)	1 (3.85)	2 (4.35)	
SCAI Class				0.994
C	14 (19.44)	5 (19.23)	9 (19.57)	
D	55 (76.39)	20 (76.92)	35 (76.09)	
E	3 (4.17)	1 (3.85)	2 (4.35)	
Heart failure etiology				0.640
Ischemic	12 (16.67)	3 (11.54)	9 (19.57)	
Non‐ischemic	58 (80.56)	22 (84.62)	36 (78.26)	
Other	2 (2.78)	1 (3.85)	1 (2.17)	
Risk factors				
Diabetes	35 (48.61)	13 (50.00)	22 (47.83)	0.859
Hypertension	51 (70.83)	19 (73.08)	32 (69.57)	0.753
Chronic kidney disease	15 (20.83)	5 (19.23)	10 (21.74)	0.801
Dialysis	4 (5.56)	1 (3.85)	3 (6.52)	0.634
Endocarditis	1 (1.39)	1 (3.85)	0 (0.00)	0.180
Cerebrovascular accident/stroke	17 (23.61)	5 (19.23)	12 (26.09)	0.511
Chronic lung disease	5 (6.94)	1 (3.85)	4 (8.70)	0.437
Smoking	30 (41.67)	10 (38.46)	20 (43.48)	0.678
Peripheral artery disease	4 (5.71)	3 (11.54)	1 (2.27)	0.107
Concomitant ECMO support	13 (18.06)	3 (11.54)	10 (21.74)	0.280
Pretransplant desensitization				
Preoperative PRA				0.585
None (0%)	58 (80.56)	22 (84.62)	36 (78.26)	
Low (1%–20%)	6 (8.33)	1 (3.85)	5 (10.87)	
Medium (21%–50%)	8 (11.11)	3 (11.54)	5 (10.87)	
High (> 50%)	0 (0.00)	0 (0.00)	0 (0.00)	
Preoperative class 1 PRA	13 (18.06)	4 (15.38)	9 (19.57)	0.658
Preoperative class 2 PRA	2 (2.78)	0 (0.00)	2 (4.35)	0.281
Impella duration	22 (12‐42)	11 (9‐13)	34 (22‐53)	**< 0.001**
Pretransplant serology				
Lactate (mmol/L)	0.7 (0.6–1.0)	0.7 (0.5–1.1)	0.7 (0.6–0.9)	0.449
Lactate dehydrogenase (U/L)	303 (254–397)	321 (258–382)	298 (252–398)	0.793
Alanine transaminase (U/L)	19 (13–33)	22 (14–38)	16 (13–30)	0.336
Aspartate transferase (U/L)	26 (20–36)	29 (18–41)	25 (20–35)	0.260
Total bilirubin (mg/dL)	0.8 (0.6–1.2)	0.9 (0.6–1.3)	0.7 (0.6–1.1)	0.266
International normalized ratio	1.3 (1.1–1.4)	1.2 (1.1–1.5)	1.3 (1.2–1.4)	0.756
Hemoglobin (g/dL)	9.8 (8.4–10.6)	10.2 (7.8–10.9)	9.5 (8.4–10.3)	0.425
White blood cell count (×10^9^/L)	7.3 (5.8–8.9)	8.4 (7.0–10.5)	6.7 (5.5–8.0)	**0.002**
Platelet count (×10^9^/L)	186 (133–244)	213 (141–253)	177 (114–217)	0.243
Creatinine (mg/dL)	1.2 (0.9–1.5)	1.0 (0.9–1.4)	1.2 (1.0–1.6)	0.125
Hemodynamics				
Mean pulmonary artery pressure (mmHg)	33 (28–38)	33 (28–40)	33 (28–37)	0.700
Pulmonary capillary wedge pressure (mmHg)	23 (19–28)	23 (18–29)	24 (19–28)	0.680
Right atrial pressure (mmHg)	11 (8–13)	11 (8–13)	11 (7–14)	0.918
Cardiac index (L/min/m^2^)	2.1 (1.8–2.5)	1.9 (1.7–2.5)	2.1 (1.8–2.5)	0.232
Systemic vascular resistance (dynes·s·cm^−5^)	1334 (1040–1622)	1509 (1261–1829)	1265 (1032–1504)	**0.033**
Peripheral vascular resistance (woods units)	2.3 (1.5–3.0)	2.8 (2.0–3.7)	2.0 (1.5–2.7)	**0.009**
SvO2 (%)	55 (49–63)	56 (50–65)	55 (49–61)	0.606
Mean arterial pressure (mmHg)	81 (75–88)	79 (74–91)	81 (75–87)	0.822
Preoperative inotrope score	2.5 (2.5–3.8)	2.5 (1.3–3.8)	2.5 (2.5–4.2)	0.199
Preoperative *p* level	7 (6–8)	7 (6–8)	8 (6–8)	0.453
Echocardiographic variables				
Right ventricular dysfunction ≥ moderate	30 (42.86)	4 (15.38)	26 (59.09)	**< 0.001**
Right ventricular dilation ≥ moderate	15 (21.43)	3 (11.54)	12 (27.27)	0.121
Mitral regurgitation ≥ moderate	26 (38.24)	5 (19.23)	21 (50.00)	0.011
Aortic regurgitation ≥ moderate	2 (3.33)	0 (0.00)	2 (5.41)	0.257
Left ventricular ejection fraction	15 (12‐18)	15 (13‐18)	13 (10‐18)	0.214

*Note:* Categorical data is expressed as *n* (%), while continuous data is expressed as median (interquartile range). Bold type denotes *p* < 0.05. Concomitant ECMO support included ECMO support at any point during Impella 5.5 support.

Abbreviation: PRA = panel reactive antibodies.

Patients in both groups were similarly optimized preoperatively. There was no difference in clinical status or end‐organ function as measured by lactate, aminotransferases, and creatinine between the two groups (all *p* > 0.05). Patients with prolonged support had a slightly lower white blood cell count (6.7 vs. 8.4, *p* = 0.002). Additionally, hemodynamic variables and inotrope requirements were similar preoperatively (Table 1). Left ventricular ejection fraction was similar between groups (15% vs. 13%, *p* = 0.214) although a smaller proportion of patients in the short duration of support cohort had right ventricular dysfunction (15% vs. 59%, *p* < 0.001). Device complication rates such as hemolysis, migration, and stroke were low and not different (*p* = 0.410) between the two cohorts (Table S2).

### 3.3 Post‐transplant Cardiac and Clinical Status

Postoperative cardiac and clinical status is presented in Table 2. Following transplantation, no difference was noted in left ventricular ejection fraction (53% vs. 55%, *p* = 0.587) between the two groups. Inotrope requirements on POD 1, 3, and 7 were also similar (all *p* > 0.05). By POD 7, both cohorts had recovered from the operation, with median lactate, aminotransferase, and creatinine levels returning to normal. Hemodynamics obtained by right heart catheterization at POD 30 were also similar (all *p* > 0.05).

**TABLE 2:** Cardiac function and clinical status post‐transplant.

Variable	Total (*n* = 72)	5.5 ≤ 14 days (*n* = 26)	5.5 > 14 days (*n* = 46)	*p* value
POD 0 echocardiographic findings				
Right ventricular dysfunction ≥ moderate	13 (18.31)	5 (19.23)	8 (17.78)	0.879
Right ventricular dilation ≥ moderate	7 (10.77)	2 (8.33)	5 (12.20)	0.628
Mitral regurgitation ≥ moderate	0 (0.00)	−	−	−
Tricuspid regurgitation ≥ moderate	8 (11.11)	2 (7.69)	6 (13.04)	0.488
Aortic regurgitation ≥ moderate	0 (0.00)	—	—	—
Left ventricular ejection fraction	55 (48–65)	53 (50–68)	55 (48–65)	0.587
POD 7 serology				
Lactate (mmol/L)	0.9 (0.6–1.2)	1.0 (0.6–1.1)	0.8 (0.6–1.2)	0.386
Alanine transaminase (U/L)	24 (18–47)	28 (21–55)	24 (15–42)	0.200
Aspartate transferase (U/L)	22 (17–30)	25 (20–33)	22 (15–30)	0.057
Total bilirubin (mg/dL)	0.7 (0.5–1.0)	0.8 (0.6–1.1)	0.6 (0.5–1.0)	0.106
International normalized ratio	1.1 (1.1–1.2)	1.1 (1.1–1.2)	1.1 (1.1–1.2)	0.851
Hemoglobin (g/dL)	9.3 (8.5–10.3)	8.9 (8.5–10.2)	9.4 (8.6–10.4)	0.139
White blood cell count (×10^9^/L)	12.2 (9.2–15.2)	13.1 (11.0–15.2)	11.3 (8.7–14.9)	0.485
Creatinine (mg/dL)	1.4 (1.0–2.0)	1.4 (1.0–1.9)	1.4 (1.0–2.0)	0.911
POD 30 hemodynamics				
Mean pulmonary artery pressure (mmHg)	21 (17–27)	19 (17–27)	23 (17–27)	0.773
Pulmonary capillary wedge pressure (mmHg)	12 (8–18)	10 (8–18)	13 (8–18)	0.453
Right atrial pressure (mmHg)	6 (4–11)	8 (4–11)	6 (4–10)	0.620
Cardiac index (L/min/m^2^)	3.0 (2.7–3.5)	3.0 (2.5–3.6)	3.1 (2.8–3.5)	0.213
Systemic vascular resistance (dynes·s·cm^−5^)	1202 (1022–1392)	1241 (1043–1437)	1159 (986–1379)	0.280
Peripheral vascular resistance (woods units)	1.6 (1.2–2.1)	1.6 (1.4–2.1)	1.6 (1.0–2.0)	0.341
SvO_2_ (%)	65 (61–71)	64 (58–72)	67 (62–70)	0.367
Mean arterial pressure (mmHg)	97 (87–018)	96 (81–107)	99 (88–109)	0.472
Inotrope score				
POD 1	2.5 (2.5–3.8)	2.5 (1.3–3.8)	2.5 (2.5–4.2)	0.184
POD 3	3.8 (2.5–6.0)	3.1 (2.5–6.0)	3.8 (2.4–6.0)	0.907
POD 7	1.25 (0.0–2.5)	1.25 (0.0–3.8)	1.25 (0.0–2.5)	0.726

*Note:* Categorical data is expressed as *n* (%) while continuous data is expressed as median (interquartile range). Bold type denotes *p* < 0.05.

### 3.4 Post‐transplant Outcomes

All post‐transplant outcomes from the index admission are presented in Table 3. Length of stay in the ICU (9 vs. 8 days, *p* = 0.930) and hospital (26 vs. 20 days, *p* = 0.316) following transplantation were similar, as were rates of complications (all *p* > 0.05). Disposition was similar between the cohorts, with 29% of all patients transitioning to inpatient rehab and 67% of all patients returning home. A total of three patients (4%) died during the index admission.

**TABLE 3:** Post‐transplant outcomes.

Variable	Total (*n* = 72)	5.5 ≤ 14 Days (*n* = 26)	5.5 > 14 days (*n* = 46)	*p* value
Ischemic time	195 (177‐213)	194 (175–220)	196 (177–211)	0.907
Concomitant kidney transplant	5 (6.94)	1 (3.95)	4 (8.70)	0.437
Index admission (post‐transplant)				
Intensive care unit duration	8 (6‐13)	9 (5–13)	8 (6–13)	0.930
Length of stay	21 (15–32)	26 (16–34)	20 (14–30)	0.316
Transfusion Requirement	40 (55.56)	15 (57.69)	25 (54.35)	0.784
ECMO	11 (15.28)	5 (19.23)	6 (13.04)	0.483
Intra‐aortic balloon pump	1 (1.39)	0 (0.00)	1 (2.17)	0.449
Additional surgery	27 (37.50)	11 (42.31)	16 (34.78)	0.526
Exploration for bleeding	9 (12.50)	4 (15.38)	5 (10.87)	0.578
Arrhythmia Cardioversion	3 (4.17)	1 (3.85)	2 (4.35)	0.919
Cardiac arrest	1 (1.39)	1 (3.85)	0 (0.00)	0.180
Re‐intubation	5 (6.94)	3 (11.54)	2 (4.35)	0.249
Tracheostomy	5 (6.94)	2 (7.69)	3 (6.52)	0.851
Chest tube for pleural effusion	9 (12.50)	3 (11.54)	6 (13.04)	0.853
Cerebrovascular accident/transient ischemic event	3 (4.17)	1 (3.85)	2 (4.35)	0.919
Gastrointestinal bleed	3 (4.17)	1 (3.85)	2 (4.35)	0.919
Dialysis	12 (16.67)	6 (23.08)	6 (13.04)	0.273
Primary graft dysfunction	11 (15.28)	5 (19.23)	6 (13.04)	0.483
Disposition				0.524
Death	3 (4.17)	2 (7.69)	1 (2.17)	
Home	48 (66.67)	17 (65.38)	31 (67.39)	
Inpatient rehab	21 (29.17)	7 (26.92)	14 (30.43)	
Follow‐up				
Duration of follow‐up	611 (255–859)	521 (242–809)	644 (264–866)	0.302
Graft rejection (≥ Grade 2)	18 (25.00)	9 (34.62)	9 (19.57)	0.157
Postoperative DSA	9 (12.50)	3 (11.54)	6 (13.04)	0.853
Postoperative Type 1 DSA	3 (4.17)	1 (3.85)	2 (4.35)	0.919
Postoperative Type 2 DSA	6 (8.33)	2 (7.69)	4 (8.70)	0.882
Death at 1‐year	3 (4.17)	2 (7.69)	1 (2.17)	0.260
Death at last follow‐up	4 (5.56)	2 (7.69)	2 (4.35)	0.552

*Note:* Categorical data is expressed as n (%) while continuous data is expressed as median (interquartile range). Bold type denotes *p* < 0.05. Primary graft dysfunction was determined based on clinical documentation.

Abbreviation: DSA = donor‐specific antibodies.

**FIGURE 1:**
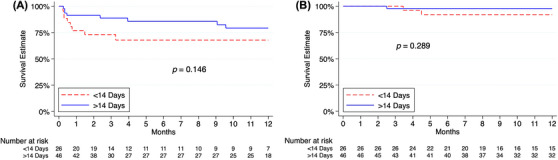
Post‐transplant time‐to‐event analysis for patients previously supported by Impella 5.5. (A) Graft Rejection. (B) Survival.

The median duration of follow‐up was 611 days following transplantation. At the time of the last follow‐up, 25% of patients had an episode of graft rejection (≥ Grade 2R ISHLT), and 12.5% of patients had positive donor‐specific antibodies (DSA); neither differed in rate when stratified by Impella 5.5 duration. The 1‐year survival free from graft rejection (Figure 1A, *p* = 0.146) and mortality (Figure 1B, *p* = 0.289) was similar.

**FIGURE 2:**
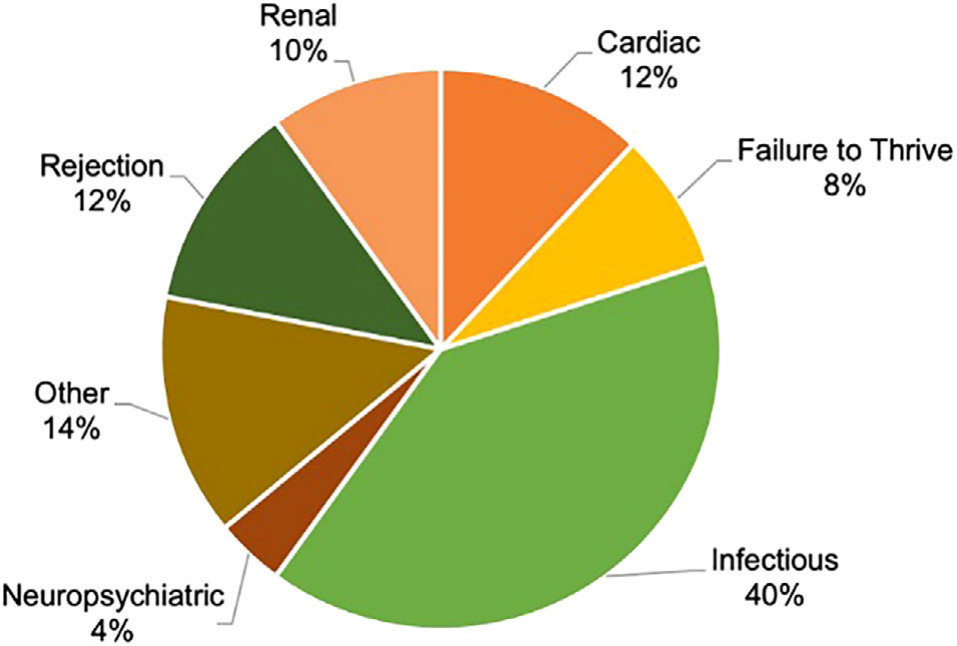
Cause of first rehospitalization.

**FIGURE 3:**
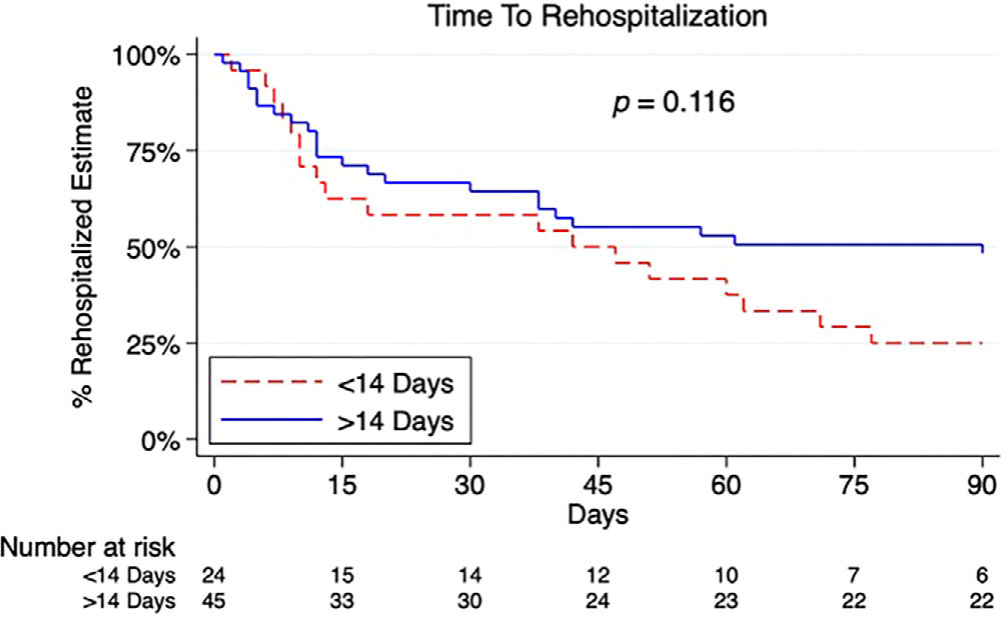
Ninety‐day survival free from rehospitalization.

All rehospitalization information for patients with 1‐year follow‐up is presented in Table 4. The most common cause for the first rehospitalization was infection (40%), followed by rejection (12%) and cardiac etiology (12%) (Figure 2). Common infectious etiologies prompting rehospitalization included pneumonia, viral prodrome, CMV viremia, and UTI, as well as two reported cases of sternal wound infections; there were no Impella surgical site infections in this cohort. 90‐day rehospitalization was not different between cohorts (Figure 3, *p* = 0.116). When all rehospitalizations in the first year were considered, patients in the shorter duration of support cohort had higher rates of rehospitalizations for cardiac issues, including arrhythmias, pericardial effusion, and clinical signs of cardiac dysfunction (33% vs. 6%, *p* = 0.015). All outcomes remain the same when patients on concomitant ECMO are excluded (Tables S3–S5).

**TABLE 4:** Rehospitalization.

Variable	Total (*n* = 47)	5.5 ≤ 14 days (*n* = 15)	5.5 > 14 days (*n* = 32)	*p* value
Rehospitalization at 1‐year	35 (74.47)	13 (86.67)	22 (68.75)	0.189
Cause of rehospitalization				
Infectious	19 (40.43)	6 (40.00)	13 (40.62)	0.968
Rejection	7 (14.89)	3 (20.00)	4 (12.50)	0.501
Cardiac	7 (14.89)	5 (33.33)	2 (6.25)	**0.015**
Renal	8 (17.02)	2 (13.33)	6 (18.75)	0.645
Neuropsychiatric	5 (10.64)	0 (0.00)	5 (15.62)	0.105
Failure to thrive	1 (2.13)	1 (6.67)	0 (0.00)	0.140
Other	10 (21.28)	5 (33.33)	5 (15.62)	0.167

*Note:* Categorical data is expressed as *n* (%). Bold type denotes *p* < 0.05. Patients with less than 1‐year follow‐up were excluded. Each hospitalization within the first year was considered.

## 4 Discussion

In this study, we examined the effect of prolonged preoperative Impella 5.5 support on heart transplant outcomes at a large tertiary medical center. The average duration of support was 22 days. Baseline characteristics were all similar between cohorts when stratified by duration of support. During the index admission, the two cohorts had similar lengths of stay and comparable dispositions. The rates of complications during the index admission and subsequent follow‐up were also similar between groups. Despite significant morbidity with a high 1‐year rehospitalization rate, the cohort overall had an excellent 1‐year survival.

Prolonged MCS support has historically been viewed as a risk factor for graft dysfunction and poor prognosis following heart transplantation. For instance, LVADs have long been associated with significant human leukocyte antigen (HLA) allosensitization [13]. Exposure of immune cells to foreign LVAD biomaterial likely results in T‐cell‐dependent B‐cell activation leading to the mass production of HLA antibodies [14]. In addition, the increased need for blood transfusions in the VAD population has also been hypothesized to increase the body's immune state prior to transplantation [15]. However, modern tMCS devices have a smaller surface area and are utilized for shorter periods of time. As such, Brow et al. found that tMCS use reduced the risk of developing HLA sensitization when compared to durable LVADs [16]. Studies specifically focused on the Impella 5.5 have also found minimal increased risk of HLA antibody development [17]. Preoperative PRA were not different in our cohorts (*p* = 0.585). Further, rates of primary graft dysfunction (19% vs. 13%, *p* = 0.483), positive DSA (12% vs. 13%, *p* = 0.853), and rejection (35% vs. 20%, *p* = 0.157)—known complications of allosensitization—in our population were not different between cohorts [18].

Vasoplegia secondary to prolonged pretransplant MCS use is another accepted predictor of primary graft dysfunction and carries its own associated morbidity [19]. The predisposition to vasoplegia may be due to blood contact with the tMCS foreign material [20]. Further, sustained support with tMCS can result in lower systemic vascular resistance and an increased risk of infection, which can additionally contribute to post‐transplant vasoplegia [20]. However, a more recent study published by Batchelor et al. found that neither duration of LVAD support nor LVAD flow requirements were predictors of post‐transplant vasoplegia [21]. Additionally, Lamba et al. did not find rates of Impella use to be different in patients with and without postoperative vasoplegia [22]. In our population, inotrope requirements were similar on POD 1, 3, and 7, suggesting no difference in vasodilation between the cohorts.

Systemic immunosuppression in transplant recipients results in a substantial infection burden. In the non‐tMCS heart transplant population, post‐transplant infections have been reported to impact between 60% and 80% of patients [23, 24]. Carrillo‐Gomez et al. reported a 39% rehospitalization rate within the first year for infectious etiologies among all heart transplant recipients at their institution—a rate closely resembling the one we report in this study [25]. Use of mechanical circulatory support platforms such as durable LVADs (2.53 [1.02–6.29]) and ECMO (14.10 [1.38–150.50]) prior to transplantation are predictors of post‐transplant infection [26]. A recent study by Trottier et al. did not find an association with tMCS use and post‐transplant infections as assessed by bacteremia, invasive fungal infections, opportunistic infections, or surgical site infections [27]. The smaller profile and shorter use of tMCS may explain the difference in effect seen between tMCS and durable LVADs. However, the impact of prolonged tMCS support has not previously been studied. In this report, no signal for increased post‐transplant infection despite prolonged tMCS support was noted. As such, continuous re‐examination of post‐transplant infections is essential as lengths of pretransplant tMCS continue to increase across institutions.

This retrospective cohort study has its limitations. As a single‐center study, the sample size is limited and may not be powered to identify differences in outcomes among patients with variable support duration. Of note, insufficient sample size precluded analysis based on heart failure etiology. Additionally, the patient population is limited to those who were successfully bridged to transplant and therefore may not be representative of all patients placed on Impella 5.5. Lastly, variable missingness in the electronic record and variable absences that are not regularly obtained for clinical care limited our analysis of the impact of prolonged Impella 5.5 on transplant outcomes.

## 5 Conclusions

In summary, we report post‐transplant outcomes for 72 patients at our institution successfully bridged to heart transplant with the Impella 5.5. We found no difference in outcomes during the index admission or at the time of last follow‐up when comparing patients based on duration of support. Our population had significant postoperative morbidity as evidenced by the prevalence of rehospitalization at 1 year following transplant, with infectious, cardiac, and renal etiologies being most common. We demonstrate that prolonged Impella 5.5 support past 14 days is not associated with increased morbidity or mortality for heart transplant recipients when compared to those supported for less than 14 days. As the heart transplantation communities consider new guidelines for graft prioritization, prolonged support should not necessarily be viewed as an indicator of poor prognosis but rather considered as a tool utilized for clinical and hemodynamic optimization prior to surgery. In fact, a recent study from the Cardiogenic Shock Working Group registry found that among 927 patients supported with the Impella 5.5, those on support for > 14 days had lower in‐hospital mortality (31% vs. 20.2%, *p* < 0.001), greater rates of heart transplantation (39% vs. 58%, *p* < 0.001), with no difference in serious adverse events (20.1% vs. 25.5%, *p* = 0.070) [28]. Since deconditioning is not as significant with modern percutaneous devices, prolonged support may be viewed as safe and potentially of benefit in certain patient populations.

## Author Contributions

David Rekhtman, Amit Iyengar, and Michael Catalano developed the research question and study design. The data were collected and analyzed by David Rekhtman, Michaela Asher, Emma Morganroth, and Alyson Brown with support from Amit Iyengar and Cindy Song. The manuscript was written by David Rekhtman and Amit Iyengar. In addition, Cindy Song, Max Shin, Michael Catalano, Omar Toubat, Aditya Parikh, Joyce Wald, Mauer Biscotti, and Marisa Cevasco provided regular support and guidance throughout the research process. All discussed and contributed to the final manuscript.

## Funding

The authors have nothing to report.

## Conflicts of Interest

Dr. Marisa Cevasco consults for and sits on the advisory board of Abiomed. The other authors declare no conflicts of interest.

## Supporting information




**Supporting Table 1:** Missingness.
**Supporting Table 2:** Impella 5.5 Complications.
**Supporting Table 3:** Cardiac Function and Clinical Status Post‐Transplant in Patients without ECMO.
**Supporting Table 4:** Post‐Transplant Outcomes in Patients without ECMO.
**Supporting Table 5:** Rehospitalization in Patients without ECMO.
**Supporting Figure 1:** Impella 5.5 Disposition.

## Data Availability

Data sharing is not applicable to this article, as no new data were created or analyzed in this study.
