# Supplementary material for: Impact of Prolonged Impella 5.5 Support on Post‐Transplant Outcomes: An Institutional Study
Source: Clin Transplant. 2026 Feb 25;40(3):e70452. doi: 10.1111/ctr.70452 (PMC12933511; doi:10.1111/ctr.70452)

**SUPPLEMENTAL**

**Supplemental Table 1:** Missingness

| Variable | Count | Percent |
| --- | --- | --- |
| Peripheral Artery Disease | 2 | 2.78 |
| Pre-Transplant Serology |  |  |
| Lactate (mmol/L) | 1 | 1.39 |
| Lactate Dehydrogenase (U/L) | 3 | 4.17 |
| Pre-Transplant Hemodynamics |  |  |
| Mean PA Pressure (mmHg) | 4 | 5.56 |
| PCWP (mmHg) | 10 | 13.89 |
| Right Atrial Pressure (mmHg) | 4 | 5.56 |
| Cardiac Index (L/min/m^2^) | 5 | 6.94 |
| SVR (dynes*sec*cm^-5^) | 8 | 11.11 |
| PVR (Woods Units) | 11 | 15.28 |
| SvO2 (%) | 5 | 6.94 |
| Mean Arterial Pressure (mmHg) | 3 | 4.17 |
| Pre-Transplant Echocardiographic Variables |  |  |
| RV Dysfunction ≥ Moderate | 2 | 2.78 |
| RV Dilation ≥ Moderate | 2 | 2.78 |
| MR ≥ Moderate | 4 | 5.56 |
| AR ≥ Moderate | 2 | 2.78 |
| POD 0 Echocardiographic Findings |  |  |
| RV Dysfunction ≥ Moderate | 1 | 1.39 |
| RV Dilation ≥ Moderate | 7 | 9.72 |
| LVEF (%) | 1 | 1.39 |
| POD 7 Serology |  |  |
| Lactate (mmol/L) | 10 | 13.89 |
| Alanine Transaminase (U/L) | 3 | 4.17 |
| Aspartate Transferase (U/L) | 3 | 4.17 |
| Total Bilirubin (mg/dL) | 3 | 4.17 |
| International Normalized Ratio | 4 | 5.56 |
| POD 30 Hemodynamics |  |  |
| Mean PA Pressure (mmHg) | 7 | 9.72 |
| PCWP (mmHg) | 8 | 11.11 |
| Right Atrial Pressure (mmHg) | 1 | 1.39 |
| Cardiac Index (L/min/m^2^) | 8 | 11.11 |
| SVR (dynes*sec*cm^-5^) | 9 | 12.50 |
| PVR (Woods Units) | 9 | 12.50 |
| SvO2 (%) | 8 | 11.11 |
| Mean Arterial Pressure (mmHg) | 5 | 6.94 |

PA = Pulmonary Artery; PCWP = Pulmonary Capillary Wedge Pressure; SVR = Systemic Vascular Resistance; PVR = Pulmonary Vascular Resistance; SvO2 = Mixed venous oxygen saturation; RV = right ventricular; MR = mitral regurgitation; AR = aortic regurgitation; LVEF = left ventricular ejection fraction; POD = postoperative day.

**Supplemental Table 2: Impella 5.5 Complications**

| Variable | Total  (n=72) | 5.5 ≤ 14 Days  (n=26) | 5.5 > 14 Days  (n=46) | P-Value |
| --- | --- | --- | --- | --- |
| Device Complications |  |  |  | 0.410 |
| Device Hemolysis* | 5 (6.94) | 3 (11.54) | 2 (4.35) |  |
| Device Migration* | 5 (6.94) | 1 (3.85) | 4 (8.70) |  |
| Purge Line Leak | 2 (2.78) | 0 (0.00) | 2 (4.35) |  |
| Ischemic Stroke | 3 (4.17) | 0 (0.00) | 3 (6.52) |  |
| Bleeding from surgical site | 1 (1.39) | 0 (0.00) | 1 (2.17) |  |
| Fiberoptic cable malfunction | 1 (1.39) | 0 (0.00) | 1 (2.17) |  |
| None | 55 (76.39) | 22 (84.62) | 33 (71.74) |  |

*Required device replacement (2 from hemolysis, 1 from migration).

**Supplemental Table 3: Cardiac Function and Clinical Status Post-Transplant in Patients without ECMO**

| Variable | Total  (n=59) | 5.5 ≤ 14 Days  (n=23) | 5.5 > 14 Days  (n=36) | P-Value |
| --- | --- | --- | --- | --- |
| POD 0 Echocardiographic Findings |  |  |  |  |
| Right Ventricular Dysfunction ≥ Moderate | 8 (13.79) | 4 (17.39) | 4 (11.43) | 0.519 |
| Right Ventricular Dilation ≥ Moderate | 5 (9.43) | 2 (9.09) | 3 (9.68) | 0.943 |
| Mitral Regurgitation ≥ Moderate | 0 (0.00) | - | - | - |
| Tricuspid Regurgitation ≥ Moderate | 5 (8.47) | 2 (8.70) | 3 (8.33) | 0.961 |
| Aortic Regurgitation ≥ Moderate | 0 (0.00) | - | - | - |
| Left Ventricular Ejection Fraction | 55 (48-65) | 53 (50-68) | 58 (48-65) | 0.923 |
| POD 7 Serology |  |  |  |  |
| Lactate (mmol/L) | 0.9 (0.6-1.2) | 0.9 (0.6-1.2) | 0.8 (0.5-1.2) | 0.485 |
| Alanine Transaminase (U/L) | 25 (15-50) | 29 (21-60) | 24 (14-42) | 0.213 |
| Aspartate Transferase (U/L) | 23 (17-30) | 25 (19-33) | 21 (15-30) | 0.079 |
| Total Bilirubin (mg/dL) | 0.6 (0.5-0.9) | 0.9 (0.6-1.1) | 0.6 (0.5-0.8) | **0.021** |
| International Normalized Ratio | 1.1 (1.1-1.2) | 1.1 (1.1-1.2) | 1.1 (1.1-1.2) | 0.902 |
| Hemoglobin (g/dL) | 9.3 (8.5-10.4) | 9.0 (8.5-10.3) | 9.4 (8.6-10.4) | 0.367 |
| White Blood Cell count (x10^9^/L) | 11.9 (9.0-15.2) | 13.2 (11.0-15.7) | 11.0 (8.4-14.7) | 0.363 |
| Creatinine (mg/dL) | 1.3 (1.0-2.0) | 1.5 (1.0-2.0) | 1.1 (1.0-2.0) | 0.529 |
| POD 30 Hemodynamics |  |  |  |  |
| Mean Pulmonary Artery Pressure (mmHg) | 20 (17-26) | 19 (17-25) | 22 (18-26) | 0.336 |
| Pulmonary Capillary Wedge Pressure (mmHg) | 11 (8-18) | 10 (8-14) | 12 (8-20) | 0.196 |
| Right Atrial Pressure (mmHg) | 6 (3-10) | 5 (3-10) | 6 (4-9) | 0.950 |
| Cardiac Index (L/min/m^2^) | 3 (2.6-3.4) | 2.9 (2.5-3.1) | 3.1 (2.9-3.4) | 0.074 |
| Systemic Vascular Resistance (dynes*sec*cm^-5^) | 1221 (1043-1386) | 1249 (1189-1454) | 1171 (1022-1353) | 0.104 |
| Peripheral Vascular Resistance (Woods Units) | 1.6 (1.2-2.0) | 1.6 (1.4-2.1) | 1.4 (1.0-2.0) | 0.337 |
| SvO2 (%) | 67 (62-71) | 63 (56-72) | 69 (64-71) | 0.160 |
| Mean Arterial Pressure (mmHg) | 98 (88-109) | 96 (79-107) | 99 (90-109) | 0.407 |
| Inotrope Score |  |  |  |  |
| POD 1 | 2.5 (2.5-3.8) | 2.5 (1.3-3.8) | 2.5 (2.5-3.8) | 0.256 |
| POD 3 | 3.8 (2.5-5.6) | 2.5 (2.5-6.0) | 3.8 (2.1-5.0) | 0.944 |
| POD 7 | 1.3 (0.0-3.0) | 1.3 (0.0-3.8) | 1.3 (0.0-2.5) | 0.701 |

Categorical data is expressed as n (%) while continuous data is expressed as median (interquartile range). Bold type denotes p<0.05.

**Supplemental Table 4: Post-Transplant Outcomes in Patients without ECMO**

| Variable | Total  (n=59) | 5.5 ≤ 14 Days  (n=23) | 5.5 > 14 Days  (n=36) | P-Value |
| --- | --- | --- | --- | --- |
| Ischemic Time | 195 (175-215) | 194 (173-222) | 196 (178-213) | 0.858 |
| Index Admission (Post Transplant) |  |  |  |  |
| Intensive Care Unit Duration | 8 (5-12) | 8 (5-13) | 7 (5-12) | 0.851 |
| Length of Stay | 19 (14-32) | 25 (13-34) | 18 (14-27) | 0.219 |
| Transfusion Requirement | 30 (50.85) | 13 (56.52) | 17 (47.22) | 0.486 |
| ECMO | 5 (8.47) | 3 (13.04) | 2 (5.56) | 0.314 |
| Intra-aortic balloon pump | 1 (1.69) | 0 (0.00) | 1 (2.78) | 0.420 |
| Additional Surgery | 19 (32.20) | 9 (39.13) | 10 (27.78) | 0.363 |
| Exploration for bleeding | 8 (13.56) | 4 (17.39) | 4 (11.11) | 0.492 |
| Arrhythmia Cardioversion | 2 (3.39) | 0 (0.00) | 2 (5.56) | 0.250 |
| Cardiac Arrest | 1 (1.69) | 1 (4.35) | 0 (0.00) | 0.207 |
| Re-intubation | 5 (8.47) | 3 (13.04) | 2 (5.56) | 0.314 |
| Tracheostomy | 5 (8.47) | 2 (8.70) | 3 (8.33) | 0.961 |
| Chest tube for pleural effusion | 7 (11.86) | 2 (8.70) | 5 (13.89) | 0.547 |
| Cerebrovascular Accident/Transient ischemic event | 3 (5.08) | 1 (4.35) | 2 (5.56) | 0.837 |
| Gastrointestinal Bleed | 3 (5.08) | 1 (4.35) | 2 (5.56) | 0.837 |
| Dialysis | 9 (15.25) | 5 (21.74) | 4 (11.11) | 0.268 |
| Primary Graft Dysfunction | 6 (10.17) | 4 (17.39) | 2 (5.56) | 0.142 |
| Disposition |  |  |  | 0.599 |
| Death | 3 (5.08) | 2 (8.70) | 1 (2.78) |  |
| Home | 43 (72.88) | 16 (69.57) | 27 (75.00) |  |
| Inpatient Rehab | 13 (22.03) | 5 (21.74) | 8 (22.22) |  |
| Follow-Up |  |  |  |  |
| Duration of Follow-Up | 496 (213-774) | 490 (191-774) | 531 (235-803) | 0.555 |
| Graft Rejection (≥ Grade 2) | 15 (25.42) | 7 (30.43) | 8 (22.22) | 0.480 |
| Postoperative DSA | 9 (15.25) | 3 (13.04) | 6 (16.67) | 0.706 |
| Postoperative Type 1 DSA | 3 (5.08) | 1 (4.35) | 2 (5.56) | 0.837 |
| Postoperative Type 2 DSA | 6 (10.17) | 2 (8.70) | 4 (11.11) | 0.765 |
| Death at 1-Year | 4 (6.78) | 2 (8.70) | 2 (5.56) | 0.640 |
| Death at Last Follow-Up | 3 (5.08) | 2 (8.70) | 1 (2.78) | 0.313 |

Categorical data is expressed as n (%) while continuous data is expressed as median (interquartile range). Bold type denotes p<0.05. Primary graft dysfunction was determined based on clinical documentation. DSA = donor specific antibodies.

**Supplemental Table 5: Rehospitalization in Patients without ECMO**

| Variable | Total  (n=35) | 5.5 ≤ 14 Days  (n=13) | 5.5 > 14 Days  (n=22) | P-Value |
| --- | --- | --- | --- | --- |
| Rehospitalization at 1-Year | 25 (71.43) | 11 (84.62) | 14 (63.64) | 0.184 |
| Cause of Rehospitalization |  |  |  |  |
| Infectious | 12 (34.29) | 5 (38.46) | 7 (31.82) | 0.689 |
| Rejection | 6 (17.14) | 3 (23.08) | 3 (13.64) | 0.474 |
| Cardiac | 7 (20.00) | 5 (38.46) | 2 (9.09) | **0.036** |
| Renal | 7 (20.00) | 2 (15.38) | 5 (22.73) | 0.600 |
| Neuropsychiatric | 2 (5.71) | 0 (0.00) | 2 (9.09) | 0.263 |
| Failure to Thrive | 1 (2.86) | 1 (7.69) | 0 (0.00) | 0.187 |
| Other | 7 (20.00) | 4 (30.77) | 3 (13.64) | 0.221 |

Categorical data is expressed as n (%). Bold type denotes p<0.05. Patients with less than 1-year follow-up were excluded. Each hospitalization within the first year was considered.

**Supplemental Figure 1:** Impella 5.5 Disposition


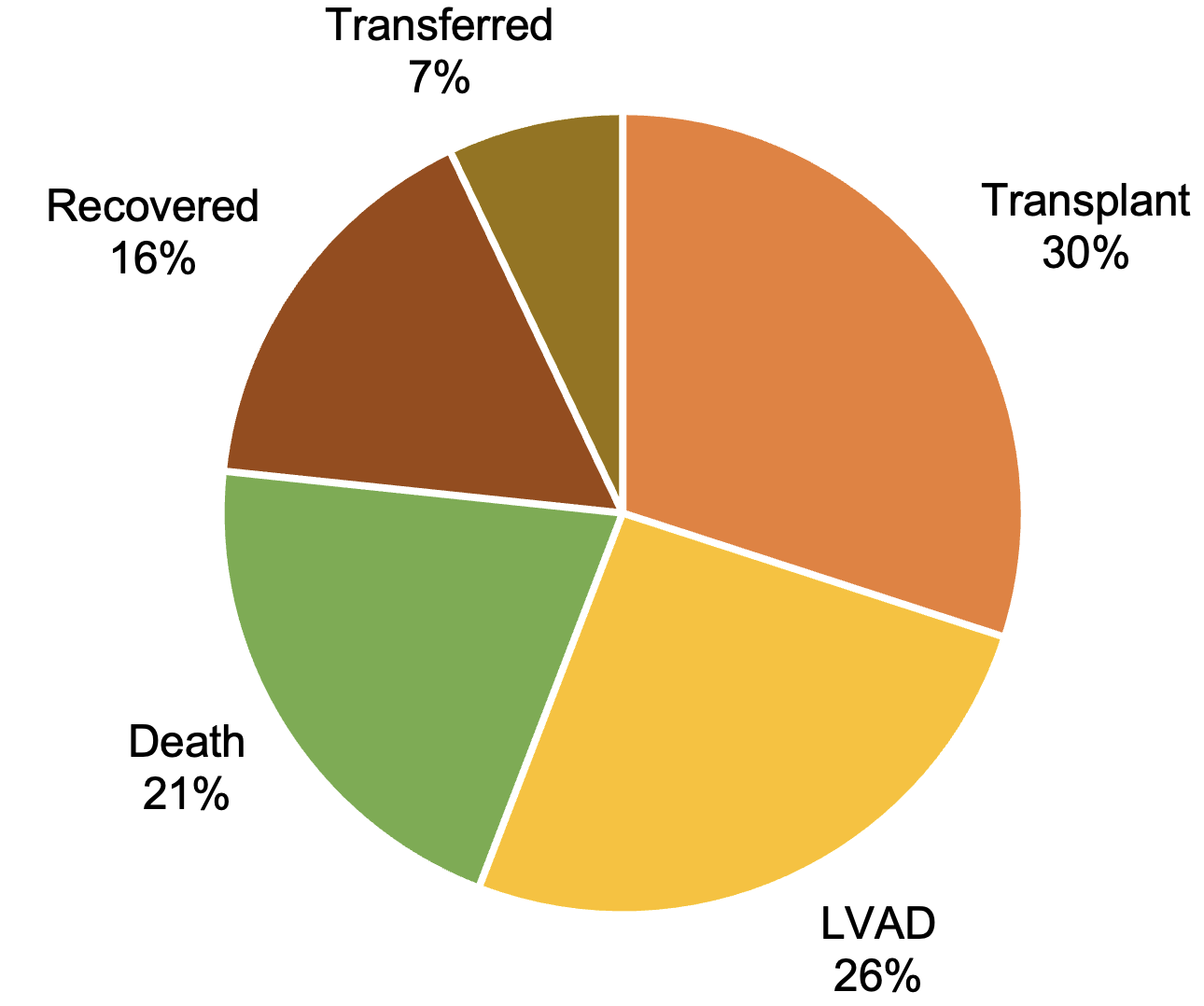

Supplement: Supplementary file 1 — Supporting Table 1: Missingness. Supporting Table 2: Impella 5.5 Complications. Supporting Table 3: Cardiac Function and Clinical Status Post‐Transplant in Patients without ECMO. Supporting Table 4: Post‐Transplant Outcomes in Patients without ECMO. Supporting Table 5: Rehospitalization in Patients without ECMO. Supporting Figure 1: Impella 5.5 Disposition. [file CTR-40-e70452-s001.docx]
